# Allosteric regulation of noncoding RNA function by microRNAs

**DOI:** 10.1093/nar/gkac443

**Published:** 2022-06-01

**Authors:** Carlos Gorbea, Abdalla Elhakiem, Demián Cazalla

**Affiliations:** Department of Biochemistry, University of Utah School of Medicine, Salt Lake City, UT 84112, USA; Department of Biochemistry, University of Utah School of Medicine, Salt Lake City, UT 84112, USA; Department of Biochemistry, University of Utah School of Medicine, Salt Lake City, UT 84112, USA

## Abstract

HSUR1 and HSUR2, two noncoding RNAs expressed by the oncogenic *Herpesvirus saimiri*, bind host microRNAs miR-142-3p, miR-16, and miR-27 with different purposes. While binding of miR-27 to HSUR1 triggers the degradation of the microRNA, miR-16 is tethered by HSUR2 to target host mRNAs to repress their expression. Here we show that the interaction with miR-142-3p is required for the activity of both HSURs. Coimmunoprecipitation experiments revealed that miR-142-3p allosterically regulates the binding of miR-27 and miR-16 to HSUR1 and HSUR2, respectively. The binding of two different miRNAs to each HSUR is not cooperative. HSURs can be engineered to be regulated by other miRNAs, indicating that the identity of the binding miRNA is not important for HSUR regulation. Our results uncover a mechanism for allosteric regulation of noncoding RNA function and a previously unappreciated way in which microRNAs can regulate gene expression.

## INTRODUCTION

The oncogenic *Herpesvirus saimiri* (HVS) establishes latency in T cells of New World primates and has the ability to transform such cells in non-natural hosts, causing lymphomas and leukemias (1). HVS encodes seven small nuclear, uracil-rich noncoding RNAs (ncRNAs) called HSURs (*Herpesvirus saimiri*U-rich RNAs). HSURs are conserved among HVS strains and in the related *Herpesvirus ateles* (HVA) (2). HSURs are the most highly abundant viral transcripts (HSUR1 is present at ∼20 000 copies per cell whereas HSUR2 is present at ∼2000 copies per cell) in latently infected, HVS-transformed cells (3–6), suggesting an important role for these viral ncRNAs in HVS latency. HSURs belong to the Sm-class family of small nuclear RNAs (snRNAs). Like the cellular snRNAs that function in pre-mRNA splicing and histone pre-mRNA 3′ end processing, HSURs bind Sm proteins and are incorporated into ribonucleoprotein particles (RNPs) through the same biogenesis pathway (7). Functions have only been assigned to HSUR1 and HSUR2. These two viral snRNAs interact with host-encoded short regulatory ncRNAs called microRNAs (miRNAs) (8). miRNAs canonically function by binding to the 3′ untranslated regions (3′UTRs) of mRNAs to promote the degradation and diminished translation of target mRNAs (9). The specificity of miRNA:target interactions is usually dictated by the seed region (nucleotides 2–7) of the miRNA. miRNA expression can be specific, with some miRNAs exhibiting high tissue-specific expression while others showing ubiquitous expression (10). One miRNA can regulate multiple mRNAs in the same pathway, and several miRNAs can act cooperatively to regulate one target mRNA (9).

**Figure 1.**
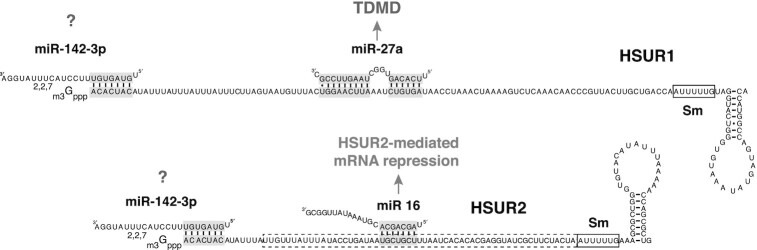
Interactions between HSUR1, HSUR2 and miRNAs. Sequences of HSUR1, HSUR2, miR-142-3p, miR-16 and miR-27 are shown. Binding sites for miRNAs and miRNA seed regions are highlighted in grey. Binding of miR-27 to HSUR1 results in TDMD (8), while miR-16 is used in HSUR2-mediated mRNA repression (17,18). Sm-binding sites are shown in black boxes. The HSUR2 region involved in interactions with mRNAs (18) is shown in a dashed box.

In HVS-infected cells, HSUR1 interacts with miR-27 and HSUR2 interacts with miR-16, whereas the 5′ end of both viral snRNAs interacts with miR-142-3p (Figure 1). A second, functional isoform of miR-142-3p that lacks the 5′ terminal U is co-expressed with miR-142-3p in lymphoid cells (11). This second isoform, or isomiR, of miR-142-3p, called miR-142-3p-1, is believed to arise from differential processing of the miR-142 primary transcript and displays a slightly different seed region. Hence, miR-142-3p-1 regulates targets through a largely discrepant set of binding sites (12). It is currently unknown if miR-142-3p-1 can also bind HSUR1 and HSUR2. These viral snRNA:miRNA interactions result in different functional outcomes. HSUR1 binds miR-27 family members in an unusual fashion. In addition to exhibiting complementarity to the seed region of miR-27, HSUR1 also exhibits extensive complementarity to the 3′ region of the miRNA (Figure 1). This kind of miRNA:target interaction results in the degradation of the miRNA by a process termed target RNA-directed miRNA degradation (TDMD) (13). The HSUR1:miR-27 interaction provided the first example of TDMD (8), a mechanism that is widely employed to regulate miRNA populations (14,15). Diminished abundance of miR-27 promotes the activation of the infected T cell and viral latency (16). The HSUR1:miR-142-3p interaction has not been experimentally validated and its functional role is unknown.

HSUR2 does not affect the abundance or activity of miR-142-3p and miR-16 (17,18). Instead, HSUR2 functions as a miRNA adaptor that utilizes miR-142-3p and miR-16 to regulate host gene expression. HSUR2 basepairs with host mRNAs and recruits these two host miRNAs to destabilize target mRNAs. It was first shown that miR-142-3p activity is required for repression of all HSUR2 target mRNAs whereas miR-16 activity is only required for the repression of a subset of target mRNAs (17). HVS utilizes HSUR2 to inhibit apoptosis in infected cells, and the activity of both miR-142-3p and miR-16 is required for HSUR2-mediated inhibition of this process (17). When HSUR2 function was first described, several mechanistic aspects remained unclear. For example, it was not understood how HSUR2 recognizes target mRNAs and why miR-142-3p is required for repression of all HSUR2 target mRNAs while miR-16 is required for repression of only a subset of targets. The identification of sequences mediating interactions between HSUR2 and target mRNAs partially answered some of these questions (18). Most HSUR2 binding sites reside in the 3′UTRs of target mRNAs, and most targets exhibit only one binding site for this viral snRNA. HSUR2 does not contain a subregion, or ‘seed sequence’, that is used for most HSUR2:mRNA interactions. Instead, an extensive region of HSUR2 (Figure 1) is involved in interactions with target mRNAs, with different base-pairing arrangements employed in each HSUR2:mRNA interaction (18). Luciferase reporter-based analyses of individual HSUR2 binding sites revealed some unexpected results. HSUR2 shows base-pairing arrangement flexibility in interactions with each target mRNA since target mRNAs can be mutagenized to interact with HSUR2 through different base-pair arrangements without losing their ability to be regulated by this viral snRNA (18). Analyses of individual HSUR2 binding sites also showed that HSUR2:mRNA base-pairing dictates the requirement of miR-16 activity for repression. In miR-16-dependent targets, the HSUR2:mRNA interaction does not involve the residues that constitute the miR-16 binding site present in HSUR2 (Figure 1). This kind of HSUR2:mRNA interaction is expected to allow the binding of miR-16 to HSUR2 and its employment in HSUR2-mediated mRNA repression. By contrast, target mRNAs for which the HSUR2:mRNA base-pair arrangement involves the miR-16 binding site present in HSUR2 are expected to prevent the binding of miR-16 to HSUR2. This group of target mRNAs are regulated by HSUR2 in a miR-16-independent manner (18), explaining why miR-16 activity is only required for repression of a subset of HSUR2 target mRNAs.

Important questions regarding the role of miR-142-3p and miR-16 in HSUR2-mediated mRNA repression remain unanswered. For instance, miR-142-3p activity is required for repression of target mRNAs through every single HSUR2 binding site, regardless of the use of miR-16. Mutagenesis of HSUR2 binding sites in target mRNAs allowed the conversion of miR-16-dependent target mRNAs into miR-16-independent, and vice versa (18). These experiments also showed that HSUR2 represses miR-16-dependent and miR-16-independent targets to the same extent, indicating that miR-142-3p and miR-16 are not used in an additive manner in HSUR2-mediated mRNA repression (18). Rather, this suggests that only one miRNA, either miR-142-3p or miR-16, is used at a time by HSUR2 to repress target mRNAs. It is therefore unclear why miR-142-3p activity is required for repression of both miR-16-dependent and miR-16-independent HSUR2 binding sites in target mRNAs.

In this manuscript, we set out to determine the role of the miR-142-3p:HSUR interaction in HSUR function. Ectopic expression of miR-142-3p in HeLa cells showed that this miRNA is absolutely required for HSUR2 and, unexpectedly, HSUR1 functions. Immunoprecipitation and mutagenesis analyses experiments demonstrated that direct interaction of miR-142-3p with HSUR1 governs the binding of miR-27 to HSUR1 and subsequent degradation of miR-27 by TDMD; the interaction of miR-142-3p with HSUR2 controls the binding of miR-16 and its use in HSUR2-mediated mRNA repression. HSUR1 and HSUR2 can be engineered to interact and be regulated by other miRNAs. These results uncover an allosteric mechanism of regulation of noncoding RNA function and an unappreciated regulatory role for miRNAs.

## MATERIALS AND METHODS

### Plasmids

For stable expression of HSURs, fragments containing the U1 promoter, HSUR1 or HSUR2, and the U1 3′-end box were generated by PCR from the pUC-U1-HSUR1 and pUC-U1-HSUR2 plasmids (19) (kindly provided by Joan Steitz) and cloned into the PspXI and XbaI sites in the pLenti CMVTRE3G eGFP Puro (Addgene #27570) to generate the pLenti-U1-HSUR1 and pLenti-U1-HSUR2 plasmids. Mutant versions of these plasmids were generated by site-directed mutagenesis and confirmed by sequencing. For transient expression of miR-27b in immunoprecipitation experiments, a fragment containing the human cytomegalovirus (CMV)-intermediate early enhancer/promoter, miR-27b primary transcript, and the polyadenylation signal of the simian virus 40 (SV40) was generated by PCR using Adeno-miR-27b (Addgene #112539) as template and inserted between the *EcoR*I and *BamH*I sites of pBluescript II SK+ (Stratagene) to generate the plasmid pBS-miR-27b.

### Cell culture and transfections

HeLa (CCL-2) and 293T/17 (CRL 11268) cells were obtained from ATCC and were grown in Dulbecco's-modified Eagle's medium (DMEM) supplemented with 10% FBS, 100 U/ml of penicillin, 100 μg/ml of streptomycin and 1 mM sodium pyruvate. All lentiviral transductions were performed in HeLa cells with supernatants generated by cotransfection of 293T/17 cells with plasmids pMD2.G (Addgene #12259), pMDLG/pRRE (Addgene #12251), pRSV-Rev (Addgene #12253) and pLenti CMVTRE3G eGFP Puro for the HeLa-Control cell line, or a lentiviral targeting vector (described above) harboring HSUR1 or HSUR2 sequences under U1’s promoter and 3′-box. Stable cell lines were generated by selection with puromycin (InvivoGen). All stable Control- and HSUR-expressing cells were grown in complete medium plus 8 μg/ml of puromycin. All cell lines were routinely tested for presence of mycoplasma.

Cells were transfected using either Lipofectamine 2000 (ThermoFisher Scientific) or Lipofectamine RNAiMax (ThermoFisher Scientific) following the manufacturer's instructions. Cells in 10-cm plates were typically transfected with 150 pmol of miRNA (Integrated DNA Technologies), 150 pmol of locked nucleic acid (LNA) miRNA inhibitors (miRCURY LNA™, Exiqon), and 10 μg of pBS-miR-27b. Cells in six-well plates were typically transfected with 25 pmol of miRNA or LNA miRNA inhibitors. In all experiments, cells were harvested ∼24 h after transfection. miRCURY LNA™ microRNA Inhibitor Negative Control A was used as Control LNA, and a miR-142-3p scrambled sequence (5′-AUGUUGUCCGUUAUUCAGUUAUG-3′) was used as Control miRNA.

### Immunoprecipitation, antibodies and northern blots

Immunoprecipitation experiments using Y12 (anti-Sm) antibodies (20) were performed as previously described (4) on extracts prepared from cells harvested from 10-cm plates using 50 μl of Protein A Dynabeads (ThermoFisher Scientific) that were previously bound to the antibody. Y12 antibodies were kindly provided by Joan Steitz. Northern blot analyses were performed as previously described (8).

### Quantitative RT-PCR

Total RNA was purified from control HeLa cells and HeLa cells constitutively expressing HSUR2 stored in TRIzol^®^ according to the manufacturer's instructions (Ambion). RNA was suspended in 85 μl of water, 10 μl of 10× DNAse reaction buffer (New England Biolabs), and 10 units (5 μl) of RNAse-free DNAse I (New England Biolabs) and incubated at 37°C for 30 min. cDNA was synthesized in 20 μl reactions from 0.9 μg of DNase I-treated total RNA using the High-Capacity cDNA Reverse Transcription Kit with MultiScribe Reverse Transcriptase and random primers (Applied Biosystems). Real-time PCR was performed in 8 μl reactions using primers at 0.5 μM and KAPA SYBR green master mix (KAPA Biosystems) in a Roche 480 Light Cycler as previously described (17,18). All reactions were performed in triplicate in each independent experiment. Relative expression of HSUR2 target mRNAs was calculated as previously described (17).

### Statistical analyses

All immunoprecipitation experiments were performed at least three times, and data presented is representative of the results obtained in all experiments performed. No statistical methods were used to predetermine sample size, nor were the experiments randomized or the investigators blinded to sample allocation during experiments and evaluation of experimental results. ‘Biological replicates’ (*n*), indicated in the figure legends, refers to the number of independent experiments performed. The number of independent experiments was chosen to allow for statistical significance. Statistical analyses were performed using Graphpad Prism 9. Two-sided *P* values of biological replicates were obtained with multiple-sample Student's *t*-tests corrected for multiple comparisons with the Holm-Sídák method (alpha = 0.05).

## RESULTS

### miR-142-3p is required for HSUR function

We previously studied HSUR2-mediated mRNA repression in lymphoid cells (17,18), which show high expression of miR-142-3p (21). To study the role of miR-142-3p in HSUR2-mediated mRNA repression we decided to ectopically express HSUR2 in cells that do not express this miRNA. We chose HeLa cells for three reasons: (i) miR-142-3p expression is not detected in HeLa cells (22); (ii) HSURs have been previously shown to be efficiently expressed and assembled into Sm-precipitable small nuclear RNPs (snRNPs) with sedimentation profiles similar to those observed for HSUR snRNPs in virally transformed T cells (23) and (iii) Hela cells can be efficiently transfected with liposome-based reagents. We also decided to constitutively express HSUR2 in these cells to avoid variability associated with transient transfections. To ensure high expression of HSUR2 as observed in latently infected T cells where the HVS genome persists as multiple copies of circular episomes (24), we constitutively expressed HSUR2 under the promoter and the 3′ end processing signal (3′-box) of U1 snRNA, which have been previously shown to robustly express HSURs (19). HeLa cells were transduced in parallel with lentiviral vectors expressing either GFP or HSUR2 to generate the control HeLa (HeLa-C) or HeLa-H2 cell lines, respectively.

**Figure 2.**
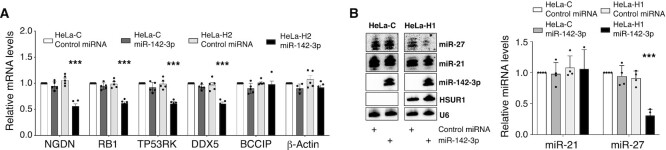
miR-142-3p is required for HSUR1 and HSUR2 activities in HeLa cells. (**A**) qRT-PCR analyses of the HSUR2 targets NGDN, RB1, TP53RK and DDX5 (17,18) and of control mRNAs encoding BCCIP and β-Actin in control HeLa cells (HeLa-C) or HeLa cells constitutively expressing HSUR2 (HeLa-H2) transiently transfected with a scrambled miR-142-3p sequence (Control) miRNA or miR-142-3p. (**B**) Northern blot analysis of HeLa-C cells and HeLa cells constitutively expressing HSUR1 (HeLa-H1) transiently transfected with Control miRNA or miR-142-3p. Right, quantification of independent experiments. U6 snRNA signal was used for normalization. For (A) and (B), dots represent mean values of independent experiments with error bars representing SD (*n* ≥ 3 per group). Statistical significance was set at *P <*0.05 and was determined with two-sided, unpaired multiple-sample Student's *t-*tests corrected with Holm–Sídák's method for multiple comparisons. **P< 0.01, ***P< 0.001.

We compared the expression of HSUR2 target mRNAs in Hela-C and Hela-H2 cells transiently transfected with a Control miRNA (miR-142-3p scrambled sequence). No changes were observed in HSUR2 target mRNAs (white and light grey bars, Figure 2A), indicating that HSUR2 alone is not active in HeLa cells. Transient expression of miR-142-3p had no effect on the levels of HSUR2 target mRNAs in HeLa-C cells, suggesting that the evaluated HSUR2 target mRNAs are not direct targets of miR-142-3p (white and dark grey bars, Figure 2A). In contrast, transient expression of miR-142-3p in HeLa-H2 cells had a significant effect on the levels of validated miR-16-dependent (RB1 and DDX5) and miR-16-independent (NGDN and TP53RK) HSUR2 target mRNAs (17,18) (black and light grey bars, Figure 2A). These results indicate that HSUR2 is active and can repress target mRNAs in HeLa cells in the presence of miR-142-3p. This is consistent with previous results showing that miR-142-3p is required for the repression of all HSUR2 target mRNAs (17,18)

HSUR1 and HSUR2 present the same sequence at their 5′ end, which is predicted to bind miR-142-3p (Figure 1). Unlike the HSUR2:miR-142-3p interaction, the interaction between HSUR1 and miR-142-3p has not been experimentally validated. Nevertheless, the perfect conservation of the miR-142-3p binding site in HSUR1 among HVS strains and the related HVA (8) suggests that the interaction with miR-142-3p is important for HSUR1 function. To test this, we generated a HeLa cell line that constitutively expresses HSUR1(HeLa-H1 cells) by transduction of a lentiviral construct expressing the HSUR1 sequence under the U1 promoter and 3′-box, as described for HSUR2. Direct comparison of miR-27 levels in HeLa-C and HeLa-H1 cells transiently transfected with Control miRNA showed that the presence of HSUR1 alone does not trigger the degradation of this miRNA, suggesting that HSUR1 is not active in HeLa cells (light grey and white bars, Figure 2B). Two distinct bands could be detected by Northern blot upon transfection of miR-142-3p mimics in HeLa cells. These could be due terminal trimming or addition of non-templated nucleotides (25). No changes in the abundance of miR-27 were observed when miR-142-3p was transiently expressed in HeLa-C cells either, indicating that miR-142-3p does not directly affect the abundance of miR-27 (dark grey and white bars, Figure 2B). In contrast, transient expression of miR-142-3p in HeLa-H1 cells resulted in lower levels of miR-27 when compared to HeLa-C cells (black and dark grey bars, Figure 2B), suggesting HSUR1 is active in the presence of miR-142-3p and can trigger degradation of miR-27 by TDMD. Altogether, these results indicate that miR-142-3p activity is required for the activities of both viral snRNAs.

### miR-142-3p regulates the binding of a second miRNA at an internal site in HSUR1 and HSUR2

HSUR1 and HSUR2 have different functions: HSUR1 promotes the degradation of a miRNA whereas HSUR2 recruits a miRNA to target mRNAs to repress their expression. However, they both need to directly interact with the miRNA (HSUR1 with miR-27 and HSUR2 with miR-16) in order to carry out their respective functions. We therefore hypothesized that miR-142-3p affects the activities of HSUR1 and HSUR2 by regulating their binding to miR-27 and miR-16, respectively. To test this, we decided to perform immunoprecipitation experiments with Y12 antibodies against Sm proteins, which HSURs bind stoichiometrically (4). miR-142-3p, miR-27, and miR-16 can be efficiently immunoprecipitated with antibodies against Sm proteins only in the presence of HSUR1 and HSUR2 (8). Detection of the HSUR1:miR-27 interaction by immunoprecipitation with Y12 antibodies and Northern blot requires long exposure times, presumably because miR-27 becomes degraded upon interaction with HSUR1 (8,26,27). To circumvent this limitation, we transiently transfected HeLa-C or HeLa-H1 cells with a plasmid expressing miR-27b. miR-27a and miR-27b sequences differ only in one residue at their 3′ end (Supplementary Figure S1). This difference is not expected to substantially affect binding of miR-27b to HSUR1 (Supplementary Figure S1) but affects the ability of miR-27b to be efficiently degraded by HSUR1-mediated TDMD (28).

**Figure 3.**
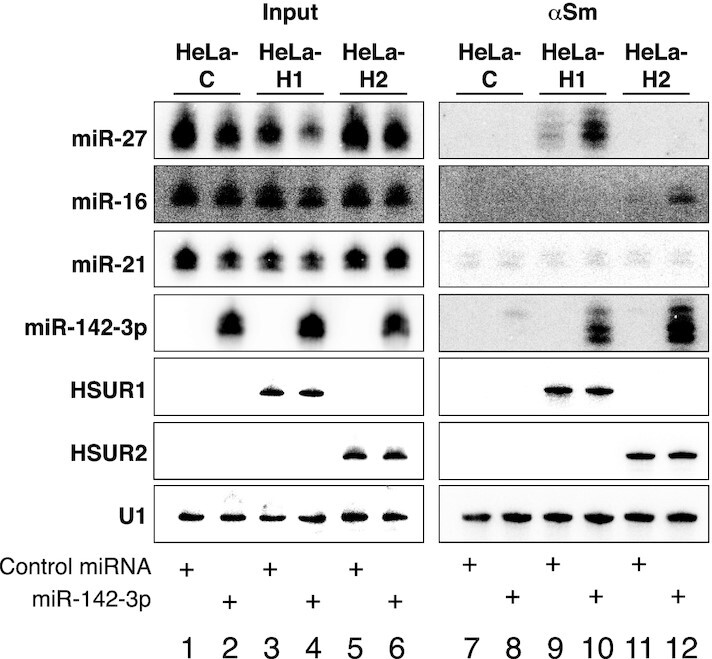
miR-142-3p regulates binding of HSUR1 and HSUR2 to a second miRNA. Coimmunoprecipitation of miRNAs from extracts prepared from equal number of HeLa-C (lanes 1, 2, 7 and 8), HeLa-H1 (lanes 3, 4, 9 and 10), or HeLa-H2 cells (lanes 5, 6, 11 and 12) transiently cotransfected with a plasmid expressing miR-27b and Control miRNA (lanes 1, 3, 5, 7, 9 and 11) or miR-142-3p (lanes 2, 4, 6, 8, 10 and 12), with Y12 (αSm) antibodies. Northern blot was probed for HSURs, miRNAs, and U1 as an immunoprecipitation control. Input: 10%, lanes 1–6; αSm: 100%, lanes 7–12. Experiment shown is representative of at least three independent experiments.

**Figure 4.**
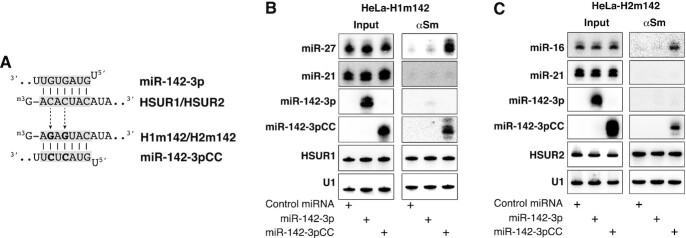
Direct interaction between miR-142-3p and HSURs is required for binding of a second miRNA. (**A**) Predicted basepairing between HSUR1 or HSUR2 and miR-142-3p, and between H1m142 or H2m142 and miR-142-3pCC. Mutated residues are shown in bold. (**B**) Same as in Figure 3 with HeLa cells expressing a mutant version of HSUR1 (HeLa-H1m142) carrying point mutations in the miR-142-3p binding site (H1m142, shown in A) transiently transfected with a plasmid expressing miR-27b and Control miRNA, miR-142-3p or miR-142-3pCC. (**C**) Same as in (B) with HeLa cells expressing a mutant version of HSUR2 (HeLa-H2m142) carrying point mutations in the miR-142-3p binding site (H2m142, shown in A) transiently transfected with Control miRNA, miR-142-3p, or miR-142-3pCC.

Immunoprecipitation experiments on extracts prepared from HeLa-C cells showed that in the absence of HSUR1 and HSUR2, Y12 antibodies against Sm proteins do not efficiently precipitate any of the miRNAs tested (Figure 3, lanes 1, 2, 7 and 8). Immunoprecipitation of Sm proteins in extracts prepared from HeLa-H1 cells transiently transfected with Control miRNA showed only background association of HSUR1 with miR-27 (Figure 3, lanes 3 and 9). In contrast, miR-142-3p and miR-27 can be both efficiently immunoprecipitated with Y12 antibodies from extracts of HeLa-H1 cells transiently transfected with miR-142-3p (Figure 3, lanes 4 and 10), suggesting that the association of miR-142-3p with HSUR1 promotes the association of miR-27 with this viral snRNA. Similar results were obtained with HSUR2. miR-16 was not efficiently immunoprecipitated with Y12 antibodies from extracts prepared from HeLa-H2 cells transiently transfected with Control miRNA (Figure 3, lanes 5 and 11). However, HSUR2 associated with both miR-16 and miR-142-3p when the latter was transiently expressed in these cells (Figure 3, lanes 6 and 12). These results suggest that binding of miR-142-3p at the 5′ end of HSUR1 and HSUR2 promotes the binding of a second miRNA at an internal, distal binding site present in these viral snRNAs. Another possibility is that the presence of miR-142-3p in HeLa cells induces changes in gene expression that could eventually modify the conformation of the HSUR1 and HSUR2 snRNPs, allowing the binding of a second miRNA. To rule out one of these two possibilities, we generated HeLa cell lines that constitutively express mutant versions of HSUR1 or HSUR2 (HeLa-H1m142 and HeLa-H2m142), each carrying two point-mutations in their 5′ ends expected to prevent binding of miR-142-3p (Figure 4A).

As shown in Figure 4B, antibodies against Sm proteins did not immunoprecipitate miR-27 nor miR-142-3p from extracts prepared from HeLa-H1m142 cells transiently transfected with miR-142-3p (Figure 4B). These results argue against the possibility that miR-142-3p indirectly induces changes in HSUR1 snRNP that allow binding of miR-27. Similar results were obtained when these experiments were performed with the HeLa-H2m142 cell line (Figure 4C). Neither miR-142-3p nor miR-16 was immunoprecipitated with Y12 antibodies in extracts prepared from HeLa-H2m142 cells transiently transfected with miR-142-3p, suggesting that miR-16 does not bind HSUR2 as a result of changes induced in HSUR2 snRNP by the presence of miR-142-3p in HeLa cells. In contrast, miR-27 associated with HSUR1 in HeLa-H1m142 cells and miR-16 associated with HSUR2 in HeLa-H2m142 cells when these cell lines were transiently transfected with a mutant version of miR-142-3p carrying compensatory mutations in the seed region predicted to restore binding to mutant HSUR1 and mutant HSUR2 (miR-142-3pCC, Figure 4B and 4C, respectively). These results strongly suggest that miR-142-3p regulates HSUR1 and HSUR2 activities by directly basepairing with these two snRNAs to promote binding of a second miRNA at an internal, distal site.

### miR-142-3p allosterically regulates the binding of HSUR1 and HSUR2 to a second miRNA

**Figure 5.**
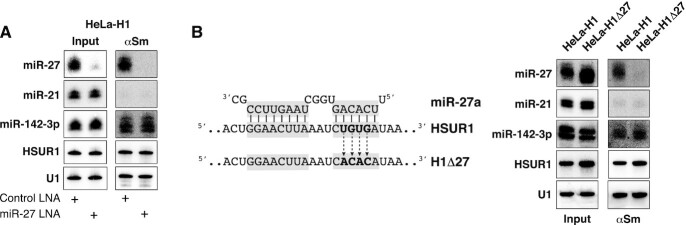
Binding of miR-142-3p to HSUR1 is independent of the binding of miR-27. (**A**) Same as in Figure 3 on extracts prepared from HeLa-H1 cells transiently cotransfected with a plasmid expressing miR-27b, miR-142-3p, and Control LNA inhibitor or an LNA inhibitor with complementarity to miR-27. (**B**) Same as in (A) on extracts prepared from equal number of HeLa-H1 cells or HeLa cells constitutively expressing a mutant version of HSUR1 that is unable to bind miR-27 (HeLa-H1Δ27) transiently cotransfected with a plasmid expressing miR-27b and miR-142-3p. Partial sequences of HSUR1 and miR-27 are shown, with residues involved in basepairing highlighted in grey. Mutated residues are shown in bold.

The results described above show that binding of miR-142-3p at the 5′ end of HSUR1 and HSUR2 correlates with binding of miR-27 and miR-16, respectively. miR-142-3p could regulate HSUR1 and HSUR2 allosterically, inducing conformational changes that allow the binding of a second miRNA at a distal site. Another alternative is that miR-142-3p and the second miRNA bind to HSUR1 and HSUR2 cooperatively. To discern between these two possibilities, we decided to block the activity of the second miRNA (miR-27 or miR-16) to test if binding of a second miRNA at a distal site is required for binding of miR-142-3p to HSUR1 and HSUR2. Transient transfection of a locked nucleic acid (LNA) inhibitor complementary to miR-27 efficiently inhibited miR-27 in HeLa-H1 cells, judged by our inability to detect this miRNA by Northern blot analyses (29) (Figure 5A, input fractions). Immunoprecipitation of HSUR1 from extracts prepared from HeLa-H1 cells transiently transfected with the miR-27 LNA inhibitor showed that disruption of the HSUR1:miR-27 interaction did not affect the HSUR1:miR-142-3p interaction since HSUR1 associated with miR-142-3p to an extent similar to that observed in HeLa-H1 cells transiently transfected with a Control LNA (Figure 5A). To confirm this result, we generated a HeLa cell line that constitutively expresses a mutant version of HSUR1 predicted to be unable to bind miR-27 since it carries four point-mutations in the sequence complementary to the seed region of miR-27 (HeLa-H1Δ27 cells, Figure 5B). This mutant version of HSUR1 does not bind miR-27, as revealed by the absence of co-immunoprecipiation of mutant HSUR1 and miR-27 with Y12 antibodies (Figure 5B). Nonetheless, mutant HSUR1 binds miR-142-3p to the same extent as wild-type HSUR1, suggesting that miR-142-3p and miR-27 do not bind cooperatively to HSUR1 and supporting a model in which miR-142-3p allosterically regulates HSUR1 function by binding to this viral snRNA to allow the binding and subsequent degradation of miR-27.

**Figure 6.**
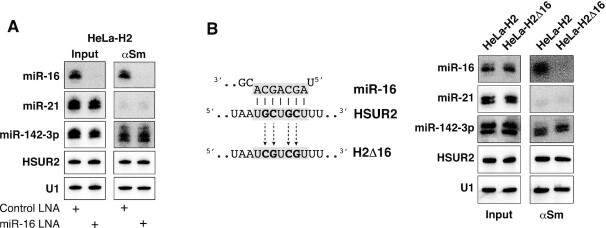
Binding of miR-142-3p to HSUR2 is independent of the binding of miR-16. (**A**) Same as in Figure 3 on extracts from HeLa-H2 cells transiently cotransfected with miR-142-3p and Control LNA inhibitor or an LNA inhibitor with complementarity to miR-16. (**B**) Same as in (A) on extracts prepared from equal number of HeLa-H2 cells or HeLa cells constitutively expressing a mutant version of HSUR2 that is unable to bind miR-16 (HeLa-H2Δ16) (17) transiently transfected with miR-142-3p. Partial sequences of HSUR2 and miR-16 are shown, with residues involved in basepairing highlighted in grey. Mutated residues are shown in bold.

Similar results were obtained for HSUR2. Blocking miR-16 activity in HeLa cells expressing HSUR2 did not affect the ability of this viral snRNA to bind miR-142-3p (Figure 6A). A mutant version of HSUR2 that does not bind miR-16 (H2Δ16; Figure 6B) represses miR-16-independent target mRNAs to the same extent as wild-type HSUR2 (17), suggesting that this mutant can still bind to miR-142-3p and utilize it for mRNA repression. Immunoprecipitation experiments confirmed that the H2Δ16 mutant version of HSUR2 binds miR-142-3p with an affinity comparable to that of wild-type HSUR2 (Figure 6B). These results indicate that miR-142-3p functions as an allosteric regulator of HSUR1 and HSUR2 that promotes binding of a second miRNA at an internal, distal site. In the case of HSUR2, miR-142-3p serves a double function, first by being tethered by HSUR2 for repression of miR-16-independent target mRNAs and second, by promoting the binding of miR-16 to HSUR2 for repression of miR-16-dependent target mRNAs (17,18).

### The identity of the miRNA is not important for allosteric regulation of HSURs

**Figure 7.**
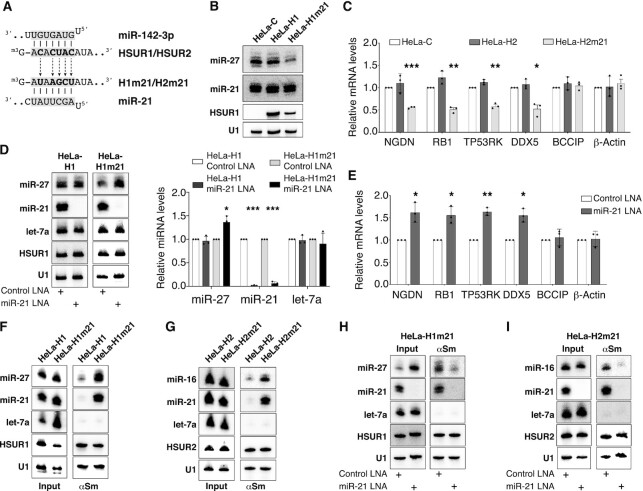
The identity of the miRNA binding at HSURs’ 5′ end is not important for allosteric regulation. (**A**) Partial sequences of the 5′ end of HSUR1 and HSUR2, and miR-142-3p and miR-21 seed regions are shown, with residues involved in basepairing highlighted in grey. Mutated residues are shown in bold. (**B**) Northern blot analysis of equal amount of total RNA prepared from HeLa-C, HeLa-H1, or HeLa cells expressing a mutant version of HSUR1 predicted to bind miR-21 at its 5′ end (HeLa-H1m21). (**C**) HSUR2 target mRNA levels in HeLa-C, HeLa-H2 and HeLa-H2m21 cells. (**D**) Same as in (B) on total RNA prepared from HeLa-H1 and HeLa-H1m21 cells transiently transfected with Control LNA inhibitor or an LNA inhibitor with complementarity to miR-21. Right, quantification of independent experiments (*n* = 3). U1 snRNA signal was used for normalization. (**E**) Same as in (C) in HeLa-H2m21 cells transiently transfected with Control LNA inhibitor or an LNA inhibitor with complementarity to miR-21. (**F**) Same as in Figure 3 on extracts prepared from equal number of HeLa-H1 or HeLa-H1m21 cells. (**G**) Same as in (F) on extracts prepared from equal number of HeLa-H2 or HeLa-H2m21 cells. (**H**) Same as in Figure 3 on extracts prepared from HeLa-H1m21 cells transiently transfected with Control LNA inhibitor or an LNA inhibitor to miR-21. (**I**) Same as in (H) on extracts prepared from HeLa-H2m21 cell.

We next wanted to test if the ability to allosterically regulate HSUR1 and HSUR2 is specific to miR-142-3p. We generated HeLa cell lines constitutively expressing mutant versions of HSUR1 and HSUR2 in which their 5′ ends were engineered to bind the seed region of miR-21 (Figure 7A), a miRNA that is abundantly expressed in HeLa cells (10,30). HeLa cells constitutively expressing a mutant version of HSUR1 predicted to bind miR-21 at its 5′ end (HeLa-H1m21) showed lower levels of miR-27 when compared to HeLa-C or HeLa-H1 cells (Figure 7B), suggesting that HSUR1 is constitutively active in these cells. Similar results were obtained with a mutant version of HSUR2 predicted to bind miR-21, since lower levels of HSUR2 target mRNAs were observed in HeLa cells constitutively expressing this mutant (HeLa-H2m21) when compared to HeLa-C or HeLa-H2 cells (Figure 7C).

Next, we tested if the mutant versions of HSUR1 and HSUR2 depended on miR-21 for their functions. Inhibition of miR-21 activity with an LNA inhibitor did not affect miR-27 levels in HeLa-H1 cells, indicating that miR-21 does not directly regulate the levels of miR-27 in HeLa cells. In contrast, inhibition of miR-21 activity resulted in higher levels of miR-27 in HeLa-H1m21 cells (black and light grey bars, Figure 7D). These results suggest that the H1m21 mutant version of HSUR1 depends on miR-21 to degrade miR-27. Similarly, miR-21 inhibition did not affect the levels of HSUR2 target mRNAs in HeLa-H2 cells (Supplementary Figure S2) indicating that miR-21 does not directly regulate the abundance of the HSUR2 target mRNAs tested but resulted in higher levels of the same mRNAs in HeLa-H2m21 cells (Figure 7E). These results suggest that these two mutant versions of HSUR1 and HSUR2 rely on miR-21 activity for their functions. Moreover, we also performed immunoprecipitation experiments to demonstrate that mutant HSUR1 and HSUR2 directly associate with miR-21 (Figure 7F, G), and that miR-21 activity is required to promote association of mutant HSUR1 and mutant HSUR2 with miR-27 or miR-16, respectively (Figure 7H, I).

Finally, similar results were obtained when HSUR1 and HSUR2 were engineered to bind let-7a (Supplementary Figure S3) or miR-122 (Supplementary Figure S4) at their 5′ end. Levels of miR-27 were constitutively lower in HeLa cells expressing a mutant version of HSUR1 that binds endogenous let-7a (HeLa-H1mlet7) relative to HeLa-H1 cells (Supplementary Figure S3B) or after transient expression of miR-122 in HeLa-H1m122 cells when compared to HeLa-H1m122 cells transfected with Control miRNA (Supplementary Figure S4B). Likewise, lower abundance of HSUR2 target mRNAs was observed in HeLa cells expressing the H2mlet7 mutant version of HSUR2 relative to HeLa-H2 cells (Supplementary Figure S3C) or in HeLa-H2m122 cells transiently expressing miR-122 when compared to HeLa-H2 or HeLa-H2m122 transiently transfected with Control miRNA (Supplementary Figure S4C). Immunoprecipitation experiments demonstrated that miR-27 or miR-16 associated with HSUR1 and HSUR2, respectively, and either let-7a or transiently expressed miR-122 in HeLa cells expressing mutant HSUR versions but not in HeLa cells expressing wild type HSUR1 or HSUR2 (Supplementary Figures S3D and 3E, S4D and 4E). Altogether, these results demonstrate that the identity of the miRNA binding at the 5′ end is not important for allosteric regulation of HSUR1 and HSUR2.

## DISCUSSION

Our results describe a previously unappreciated way in which miRNAs can regulate gene expression. The activities of HSUR1 and HSUR2 are allosterically regulated by a miRNA. The binding of miR-27 or miR-16 at an internal binding site, which is crucial for HSUR1 and HSUR2 functions, is dependent on the binding of a miRNA at the 5′ end of these snRNAs (Figures 3, 4 and 7, and Supplementary Figures S2–S4). miRNAs can act cooperatively in mRNA repression (31,32) and they have also been proposed to bind cooperatively to other classes of ncRNAs (33). However, the binding of miR-142-3p, miR-27 and miR-16 to HSUR1 and HSUR2 is not cooperative since disruption of the interaction of either of these viral snRNAs with miR-27 or miR-16 does not affect the binding of miR-142-3p at their 5′ end (Figures 5 and 6). The type of regulation exerted by miRNAs on HSUR1 and HSUR2 is better described by the first statement of allostery (34) that postulates that two distinct sites within an allosteric protein (HSUR1/HSUR2), each binding different ligands (miR-142-3p and miR-27/miR-16) could interact despite being presumably distant from each other. We speculate that binding of a miRNA at the 5′ end of HSUR1 and HSUR2 results in a conformational alteration of these snRNAs that allows the binding of the second miRNA at an internal site. It is difficult to hypothesize at this point about which structural changes in HSUR1 and HSUR2 could lead to binding of miR-27 or miR-16, respectively. Structural *in vivo* mapping of HSUR1 with dimethyl sulfate (DMS) revealed that HSUR1 is loosely structured and dynamic (26). No structural information for HSUR2 is available. Further investigation will be required to determine structural changes induced by miR-142-3p binding to these two viral snRNAs. Unlike other allosteric or cooperative regulatory systems, e.g. riboswitches (35), in which an RNA molecule binds other types of molecules, miRNA-mediated regulation of HSUR1 and HSUR2 relies solely on RNA-RNA interactions among ncRNAs.

Functional interactions between miRNAs and other classes of ncRNAs, including circular RNAs (circRNAs) and long ncRNAs (lncRNAs), have been documented (14,33,36–43). However, in most cases these interactions result in the reduction of miRNA abundance by TDMD or inhibition of miRNA activity by sequestration. Nevertheless, noncanonical functions have been ascribed to miRNAs as regulators of gene expression. Similar to HVS, Hepatitis C Virus (HCV) relies on a host-encoded miRNA for its regulation. The 5′ end of the HCV genome displays two binding sites for miR-122, a miRNA that is abundantly expressed in liver (44,45). Interaction of miR-122 with these sites substantially increases the abundance of HCV RNA (46) through still incompletely understood mechanisms (47). Another example is provided by miR-10a. This miRNA interacts with the 5′ UTR of mRNAs encoding ribosomal proteins to enhance their translation (48). These examples raise the possibility that, in addition to their canonical roles as negative regulators of gene expression when they bind mRNA 3′UTRs, miRNAs have more diverse roles as regulators of RNA-based mechanisms than previously appreciated.

HSUR1 and HSUR2 could be regulated by all miRNAs tested (Figure 7 and Supplementary Figures S3 and S4), indicating that allosteric regulation occurs regardless of the identity of the miRNA binding at the 5′ end of these viral snRNAs. However, both HSUR1 and HSUR2 evolved to bind the same miRNA, miR-142-3p, at their 5′ end. Expression of this miRNA is limited to hematopoietic tissues, where is among the most highly expressed miRNAs (10,21) and has a crucial role in hematopoietic lineage development and differentiation (49–51). HSUR1 is ∼10 times more abundant than miR-142-3p whereas HSUR2 shows similar abundance to this miRNA (4,17). However, <5% of either HSUR1 or HSUR2 associate with Ago proteins in virally transformed T cells (8), suggesting that only a small fraction of these two viral snRNAs needs to be allosterically regulated by miR-142-3p. Similar to HCV, which evolved to bind a miRNA that accounts for 70% of the total miRNA population in liver cells (44,45), HSUR1 and HSUR2 evolved to be controlled by a miRNA that is highly abundant in the cells where these snRNAs need to function. HSURs are conserved in all subgroups of HVS and abundantly expressed in latently infected T cells (4,6,52), suggesting that their functions are important in the maintenance of latency. Since HSUR1 and HSUR2 cannot function in the absence of miR-142-3p, it is conceivable that the selection of this miRNA might contribute to restrict latent HVS infections to T cells.

It is unclear why HSUR1 and HSUR2 are allosterically regulated. It is also unclear when and where HSUR2 binds to target mRNAs. Since HSUR2 is mostly nuclear (53), it is conceivable that HSUR2 recognizes and binds to target mRNAs in the nucleus and is exported to the cytoplasm bound to target mRNAs. Indeed, HSUR2 associates with translationally active mRNAs in the cytoplasm (17) as it has been previously reported for miRNAs (54,55). All miR-16 family members share the same seed sequence, and therefore can bind to HSUR2 (Figure 1). All members of the miR-16 family are also found in the cell nucleus (56–58). It is conceivable that miR-16 family members could interfere in the nucleus with the recognition of target mRNAs that basepair with the miR-16 binding site present in HSUR2. In the case of HSUR2, an allosteric regulatory mechanism as the one described here could prevent miR-16 family members from binding HSUR2 in the nucleus and therefore interfere with target mRNA recognition. Once HSUR2 is in the cytoplasm, binding of miR-142-3p would allow binding of miR-16 family members to HSUR2 if the miR-16 binding site is available. In support of this hypothesis, we have shown that base-pairing between HSUR2 and target mRNAs determines the use of miR-16 in HSUR2-mediated mRNA repression (18), suggesting that miR-16 binds to HSUR2 after the HSUR2:mRNA interaction has been established. Further investigation is required to explore this possibility.

## DATA AVAILABILITY

All data are available in the manuscript or the supplementary materials.

## Supplementary Material

gkac443_Supplemental_File

## References

[1] Ensser A. , Fleckenstein B. T-cell transformation and oncogenesis by gamma2-herpesviruses. Adv. Cancer. Res. 2005; 93:91–128. 15797445 10.1016/S0065-230X(05)93003-0

[2] Albrecht J.C. Primary structure of the Herpesvirus ateles genome. J. Virol. 2000; 74:1033–1037. 10623770 10.1128/jvi.74.2.1033-1037.2000 PMC111628

[3] Albrecht J.C. , Fleckenstein B. Nucleotide sequence of HSUR 6 and HSUR 7, two small RNAs of herpesvirus saimiri. Nucleic Acids Res. 1992; 20:1810. 1315960 10.1093/nar/20.7.1810 PMC312282

[4] Lee S.I. , Murthy S.C., Trimble J.J., Desrosiers R.C., Steitz J.A. Four novel U RNAs are encoded by a herpesvirus. Cell. 1988; 54:599–607. 2842058 10.1016/s0092-8674(88)80004-7

[5] Murthy S. , Kamine J., Desrosiers R.C. Viral-encoded small RNAs in herpes virus saimiri induced tumors. EMBO J. 1986; 5:1625–1632. 2427336 10.1002/j.1460-2075.1986.tb04405.x PMC1166988

[6] Wassarman D.A. , Lee S.I., Steitz J.A. Nucleotide sequence of HSUR 5 RNA from herpesvirus saimiri. Nucleic Acids Res. 1989; 17:1258. 2537954 10.1093/nar/17.3.1258 PMC331759

[7] Golembe T.J. , Yong J., Battle D.J., Feng W., Wan L., Dreyfuss G. Lymphotropic Herpesvirus saimiri uses the SMN complex to assemble Sm cores on its small RNAs. Mol. Cell. Biol. 2005; 25:602–611. 15632062 10.1128/MCB.25.2.602-611.2005 PMC543424

[8] Cazalla D. , Yario T., Steitz J.A. Down-regulation of a host microRNA by a Herpesvirus saimiri noncoding RNA. Science. 2010; 328:1563–1566. 20558719 10.1126/science.1187197 PMC3075239

[9] Bartel D.P. Metazoan MicroRNAs. Cell. 2018; 173:20–51. 29570994 10.1016/j.cell.2018.03.006 PMC6091663

[10] Landgraf P. , Rusu M., Sheridan R., Sewer A., Iovino N., Aravin A., Pfeffer S., Rice A., Kamphorst A.O., Landthaler M. et al. A mammalian microRNA expression atlas based on small RNA library sequencing. Cell. 2007; 129:1401–1414. 17604727 10.1016/j.cell.2007.04.040 PMC2681231

[11] Gottwein E. , Corcoran D.L., Mukherjee N., Skalsky R.L., Hafner M., Nusbaum J.D., Shamulailatpam P., Love C.L., Dave S.S., Tuschl T. et al. Viral microRNA targetome of KSHV-infected primary effusion lymphoma cell lines. Cell Host Microbe. 2011; 10:515–526. 22100165 10.1016/j.chom.2011.09.012 PMC3222872

[12] Manzano M. , Forte E., Raja A.N., Schipma M.J., Gottwein E. Divergent target recognition by coexpressed 5′-isomiRs of miR-142-3p and selective viral mimicry. RNA. 2015; 21:1606–1620. 26137849 10.1261/rna.048876.114 PMC4536321

[13] de la Mata M. , Gaidatzis D., Vitanescu M., Stadler M.B., Wentzel C., Scheiffele P., Filipowicz W., Grosshans H. Potent degradation of neuronal miRNAs induced by highly complementary targets. EMBO Rep. 2015; 16:500–511. 25724380 10.15252/embr.201540078 PMC4388616

[14] Li L. , Sheng P., Li T., Fields C.J., Hiers N.M., Wang Y., Li J., Guardia C.M., Licht J.D., Xie M. Widespread microRNA degradation elements in target mRNAs can assist the encoded proteins. Genes Dev. 2021; 35:1595–1609. 34819352 10.1101/gad.348874.121 PMC8653786

[15] Simeone I. , Rubolino C., Noviello T.M.R., Farinello D., Cerulo L., Marzi M.J., Nicassio F. Prediction and pan-cancer analysis of mammalian transcripts involved in target directed miRNA degradation. Nucleic Acids Res. 2022; 50:2019–2035. 35137158 10.1093/nar/gkac057 PMC8887481

[16] Guo Y.E. , Riley K.J., Iwasaki A., Steitz J.A. Alternative capture of noncoding RNAs or protein-coding genes by herpesviruses to alter host T cell function. Mol. Cell. 2014; 54:67–79. 24725595 10.1016/j.molcel.2014.03.025 PMC4039351

[17] Gorbea C. , Mosbruger T., Cazalla D. A viral Sm-class RNA base-pairs with mRNAs and recruits microRNAs to inhibit apoptosis. Nature. 2017; 550:275–279. 28976967 10.1038/nature24034 PMC5864290

[18] Gorbea C. , Mosbruger T., Nix D.A., Cazalla D. Viral miRNA adaptor differentially recruits miRNAs to target mRNAs through alternative base-pairing. Elife. 2019; 8:e50530. 31538617 10.7554/eLife.50530 PMC6763288

[19] Fan X.C. , Myer V.E., Steitz J.A. AU-rich elements target small nuclear RNAs as well as mRNAs for rapid degradation. Genes Dev. 1997; 11:2557–2568. 9334320 10.1101/gad.11.19.2557 PMC316563

[20] Lerner E.A. , Lerner M.R., Janeway C.A., Steitz J.A Monoclonal antibodies to nucleic acid-containing cellular constituents: probes for molecular biology and autoimmune disease. Proc. Natl. Acad. Sci. U.S.A. 1981; 78:2737–2741. 6789322 10.1073/pnas.78.5.2737 PMC319432

[21] Chen C.Z. , Li L., Lodish H.F., Bartel D.P. MicroRNAs modulate hematopoietic lineage differentiation. Science. 2004; 303:83–86. 14657504 10.1126/science.1091903

[22] Lv M. , Zhang X., Jia H., Li D., Zhang B., Zhang H., Hong M., Jiang T., Jiang Q., Lu J. et al. An oncogenic role of miR-142-3p in human T-cell acute lymphoblastic leukemia (T-ALL) by targeting glucocorticoid receptor-alpha and cAMP/PKA pathways. Leukemia. 2012; 26:769–777. 21979877 10.1038/leu.2011.273

[23] Lee S.I. , Steitz J.A. Herpesvirus saimiri U RNAs are expressed and assembled into ribonucleoprotein particles in the absence of other viral genes. J. Virol. 1990; 64:3905–3915. 2164602 10.1128/jvi.64.8.3905-3915.1990 PMC249686

[24] Biesinger B. , Muller-Fleckenstein I., Simmer B., Lang G., Wittmann S., Platzer E., Desrosiers R.C., Fleckenstein B. Stable growth transformation of human T lymphocytes by herpesvirus saimiri. Proc. Natl. Acad. Sci. U.S.A. 1992; 89:3116–3119. 1313581 10.1073/pnas.89.7.3116 PMC48815

[25] Ameres S.L. , Zamore P.D. Diversifying microRNA sequence and function. Nat. Rev. Mol. Cell Biol. 2013; 14:475–488. 23800994 10.1038/nrm3611

[26] Pawlica P. , Moss W.N., Steitz J.A. Host miRNA degradation by Herpesvirus saimiri small nuclear RNA requires an unstructured interacting region. RNA. 2016; 22:1181–1189. 27335146 10.1261/rna.054817.115 PMC4931111

[27] Shi C.Y. , Kingston E.R., Kleaveland B., Lin D.H., Stubna M.W., Bartel D.P. The ZSWIM8 ubiquitin ligase mediates target-directed microRNA degradation. Science. 2020; 370:1563–1566. 10.1126/science.abc9359 PMC8356967 33184237

[28] Sheu-Gruttadauria J. , Pawlica P., Klum S.M., Wang S., Yario T.A., Schirle Oakdale N.T., Steitz J.A., MacRae I.J. Structural basis for target-directed MicroRNA degradation. Mol. Cell. 2019; 75:1243–1255. 31353209 10.1016/j.molcel.2019.06.019 PMC6754277

[29] Torres A.G. , Fabani M.M., Vigorito E., Gait M.J. MicroRNA fate upon targeting with anti-miRNA oligonucleotides as revealed by an improved Northern-blot-based method for miRNA detection. RNA. 2011; 17:933–943. 21441346 10.1261/rna.2533811 PMC3078742

[30] Nelson P.T. , Hatzigeorgiou A.G., Mourelatos Z. miRNP:mRNA association in polyribosomes in a human neuronal cell line. RNA. 2004; 10:387–394. 14970384 10.1261/rna.5181104 PMC1370934

[31] Grimson A. , Farh K.K., Johnston W.K., Garrett-Engele P., Lim L.P., Bartel D.P. MicroRNA targeting specificity in mammals: determinants beyond seed pairing. Mol. Cell. 2007; 27:91–105. 17612493 10.1016/j.molcel.2007.06.017 PMC3800283

[32] Saetrom P. , Heale B.S., Snove O., Aagaard L., Alluin J., Rossi J.J Distance constraints between microRNA target sites dictate efficacy and cooperativity. Nucleic Acids Res. 2007; 35:2333–2342. 17389647 10.1093/nar/gkm133 PMC1874663

[33] Kleaveland B. , Shi C.Y., Stefano J., Bartel D.P. A network of noncoding regulatory RNAs acts in the mammalian brain. Cell. 2018; 174:350–362. 29887379 10.1016/j.cell.2018.05.022 PMC6559361

[34] Monod J. , Changeux J.P., Jacob F. Allosteric proteins and cellular control systems. J. Mol. Biol. 1963; 6:306–329. 13936070 10.1016/s0022-2836(63)80091-1

[35] Peselis A. , Gao A., Serganov A. Cooperativity, allostery and synergism in ligand binding to riboswitches. Biochimie. 2015; 117:100–109. 26143008 10.1016/j.biochi.2015.06.028 PMC4643686

[36] Bian E.B. , Ma C.C., He X.J., Wang C., Zong G., Wang H.L., Zhao B. Epigenetic modification of miR-141 regulates SKA2 by an endogenous ‘sponge’ HOTAIR in glioma. Oncotarget. 2016; 7:30610–30625. 27121316 10.18632/oncotarget.8895 PMC5058705

[37] Bitetti A. , Mallory A.C., Golini E., Carrieri C., Carreno Gutierrez H., Perlas E., Perez-Rico Y.A., Tocchini-Valentini G.P., Enright A.J., Norton W.H.J. et al. MicroRNA degradation by a conserved target RNA regulates animal behavior. Nat. Struct. Mol. Biol. 2018; 25:244–251. 29483647 10.1038/s41594-018-0032-x

[38] Franco-Zorrilla J.M. , Valli A., Todesco M., Mateos I., Puga M.I., Rubio-Somoza I., Leyva A., Weigel D., Garcia J.A., Paz-Ares J. Target mimicry provides a new mechanism for regulation of microRNA activity. Nat. Genet. 2007; 39:1033–1037. 17643101 10.1038/ng2079

[39] Hansen T.B. , Jensen T.I., Clausen B.H., Bramsen J.B., Finsen B., Damgaard C.K., Kjems J. Natural RNA circles function as efficient microRNA sponges. Nature. 2013; 495:384–388. 23446346 10.1038/nature11993

[40] Imig J. , Brunschweiger A., Brummer A., Guennewig B., Mittal N., Kishore S., Tsikrika P., Gerber A.P., Zavolan M., Hall J. miR-CLIP capture of a miRNA targetome uncovers a lincRNA H19-miR-106a interaction. Nat. Chem. Biol. 2015; 11:107–114. 25531890 10.1038/nchembio.1713

[41] Liu S. , Song L., Zeng S., Zhang L. MALAT1-miR-124-RBG2 axis is involved in growth and invasion of HR-HPV-positive cervical cancer cells. Tumour Biol. 2016; 37:633–640. 26242259 10.1007/s13277-015-3732-4

[42] Memczak S. , Jens M., Elefsinioti A., Torti F., Krueger J., Rybak A., Maier L., Mackowiak S.D., Gregersen L.H., Munschauer M. et al. Circular RNAs are a large class of animal RNAs with regulatory potency. Nature. 2013; 495:333–338. 23446348 10.1038/nature11928

[43] Wang J. , Liu X., Wu H., Ni P., Gu Z., Qiao Y., Chen N., Sun F., Fan Q. CREB up-regulates long non-coding RNA, HULC expression through interaction with microRNA-372 in liver cancer. Nucleic Acids Res. 2010; 38:5366–5383. 20423907 10.1093/nar/gkq285 PMC2938198

[44] Chang J. , Nicolas E., Marks D., Sander C., Lerro A., Buendia M.A., Xu C., Mason W.S., Moloshok T., Bort R. et al. miR-122, a mammalian liver-specific microRNA, is processed from hcr mRNA and may downregulate the high affinity cationic amino acid transporter CAT-1. RNA Biol. 2004; 1:106–113. 17179747 10.4161/rna.1.2.1066

[45] Lagos-Quintana M. , Rauhut R., Yalcin A., Meyer J., Lendeckel W., Tuschl T. Identification of tissue-specific microRNAs from mouse. Curr. Biol. 2002; 12:735–739. 12007417 10.1016/s0960-9822(02)00809-6

[46] Jopling C.L. , Yi M., Lancaster A.M., Lemon S.M., Sarnow P. Modulation of hepatitis C virus RNA abundance by a liver-specific MicroRNA. Science. 2005; 309:1577–1581. 16141076 10.1126/science.1113329

[47] Kunden R.D. , Khan J.Q., Ghezelbash S., Wilson J.A. The role of the liver-specific microRNA, miRNA-122 in the HCV replication cycle. Int. J. Mol. Sci. 2020; 21:5677. 10.3390/ijms21165677 PMC7460827 32784807

[48] Orom U.A. , Nielsen F.C., Lund A.H. MicroRNA-10a binds the 5′UTR of ribosomal protein mRNAs and enhances their translation. Mol. Cell. 2008; 30:460–471. 18498749 10.1016/j.molcel.2008.05.001

[49] Kramer N.J. , Wang W.L., Reyes E.Y., Kumar B., Chen C.C., Ramakrishna C., Cantin E.M., Vonderfecht S.L., Taganov K.D., Chau N. et al. Altered lymphopoiesis and immunodeficiency in miR-142 null mice. Blood. 2015; 125:3720–3730. 25931583 10.1182/blood-2014-10-603951

[50] Lu X. , Li X., He Q., Gao J., Gao Y., Liu B., Liu F. miR-142-3p regulates the formation and differentiation of hematopoietic stem cells in vertebrates. Cell Res. 2013; 23:1356–1368. 24165894 10.1038/cr.2013.145 PMC3847575

[51] Nimmo R. , Ciau-Uitz A., Ruiz-Herguido C., Soneji S., Bigas A., Patient R., Enver T. MiR-142-3p controls the specification of definitive hemangioblasts during ontogeny. Dev. Cell. 2013; 26:237–249. 23911199 10.1016/j.devcel.2013.06.023

[52] Biesinger B. , Trimble J.J., Desrosiers R.C., Fleckenstein B. The divergence between two oncogenic Herpesvirus saimiri strains in a genomic region related to the transforming phenotype. Virology. 1990; 176:505–514. 2161148 10.1016/0042-6822(90)90020-r

[53] Chou C.S. , Medveczky M.M., Geck P., Vercelli D., Medveczky P.G. Expression of IL-2 and IL-4 in T lymphocytes transformed by herpesvirus saimiri. Virology. 1995; 208:418–426. 7747414 10.1006/viro.1995.1172

[54] Maroney P.A. , Yu Y., Fisher J., Nilsen T.W. Evidence that microRNAs are associated with translating messenger RNAs in human cells. Nat. Struct. Mol. Biol. 2006; 13:1102–1107. 17128271 10.1038/nsmb1174

[55] Nottrott S. , Simard M.J., Richter J.D. Human let-7a miRNA blocks protein production on actively translating polyribosomes. Nat. Struct. Mol. Biol. 2006; 13:1108–1114. 17128272 10.1038/nsmb1173

[56] Ashe M.P. , Griffin P., James W., Proudfoot N.J. Poly(A) site selection in the HIV-1 provirus: inhibition of promoter-proximal polyadenylation by the downstream major splice donor site. Genes Dev. 1995; 9:3008–3025. 7498796 10.1101/gad.9.23.3008

[57] Chu Y. , Yokota S., Liu J., Kilikevicius A., Johnson K.C., Corey D.R. Argonaute binding within human nuclear RNA and its impact on alternative splicing. RNA. 2021; 27:991–1003. 34108230 10.1261/rna.078707.121 PMC8370746

[58] Liao J.Y. , Ma L.M., Guo Y.H., Zhang Y.C., Zhou H., Shao P., Chen Y.Q., Qu L.H. Deep sequencing of human nuclear and cytoplasmic small RNAs reveals an unexpectedly complex subcellular distribution of miRNAs and tRNA 3′ trailers. PLoS One. 2010; 5:e10563. 20498841 10.1371/journal.pone.0010563 PMC2871053

