# Supplementary material for: Allosteric regulation of noncoding RNA function by microRNAs
Source: Nucleic Acids Res. 2022 Jun 1;50(11):6511–20. doi: 10.1093/nar/gkac443 (PMC9226524; doi:10.1093/nar/gkac443)
Supplement: gkac443_Supplemental_File [file gkac443_supplemental_file.docx]

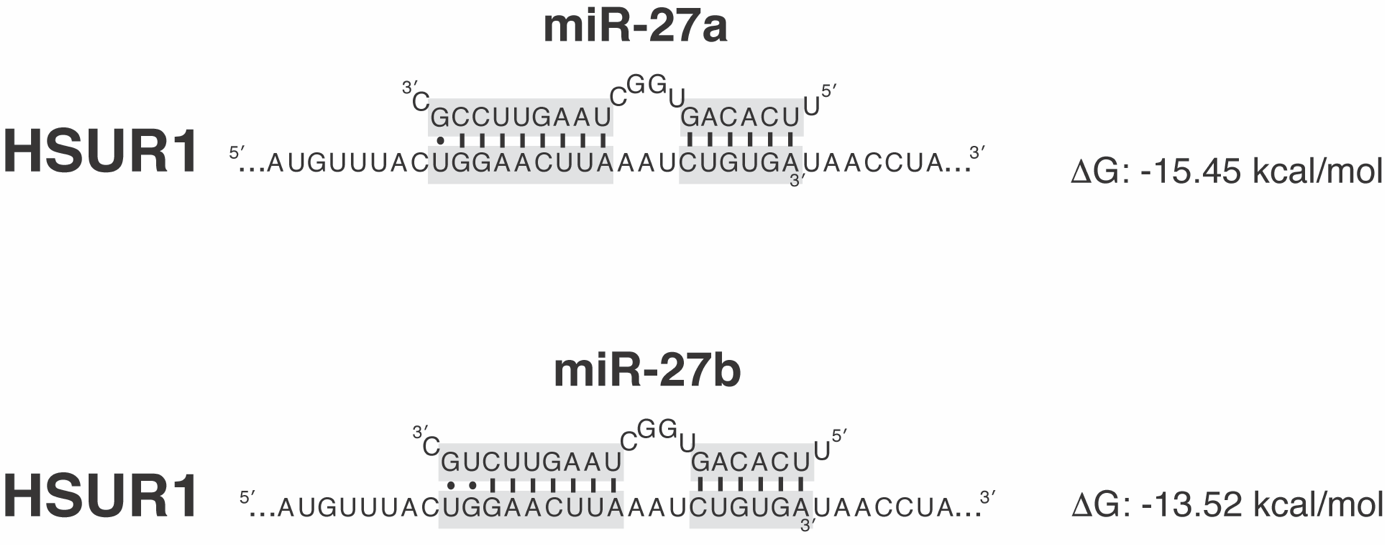


**Supplementary Figure 1. HSUR1:miR-27 interactions**.

Partial sequence of HSUR1 and either miR-27a or miR-27b are shown, with residues involved in basepairing highlighted in gray. The ΔG for heterodimer binding was calculated using the RNAcofold web server from the ViennaRNA Package 2.0 at the Institute of Theoretical Chemistry at the University of Vienna (<http://rna.tbi.univie.ac/at/RNAcofold>).


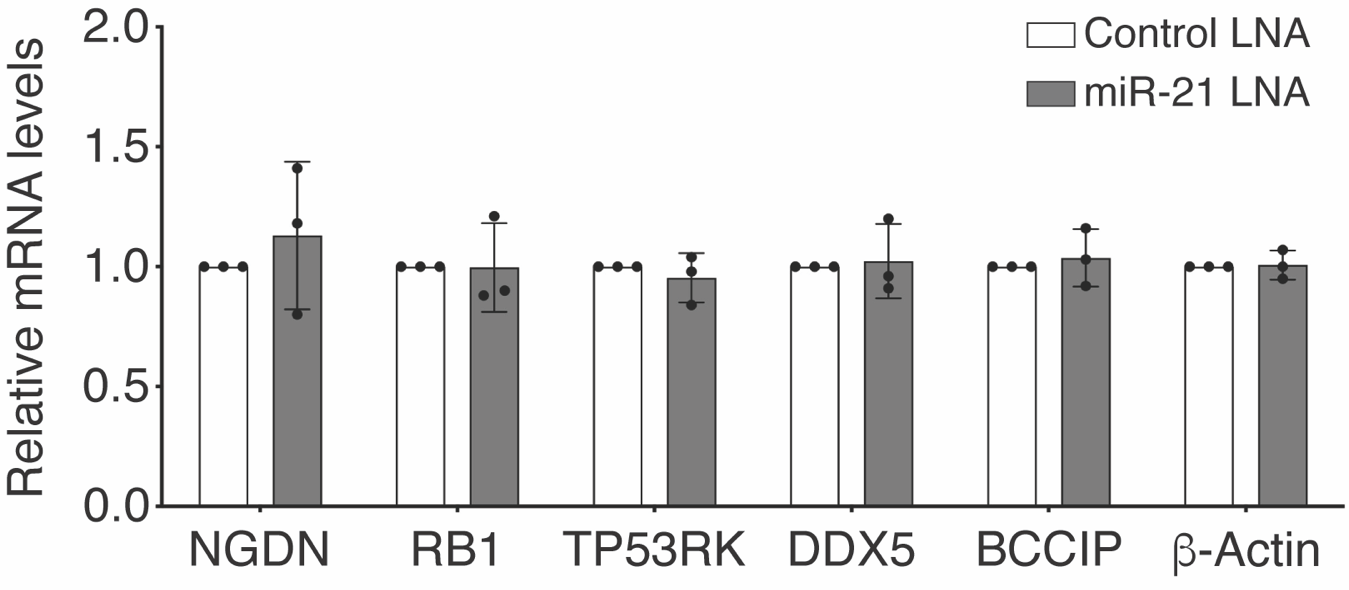


**Supplementary Figure 2. The identity of the miRNA binding at HSURs’ 5′ end is not important for allosteric regulation.**

Same as in Fig. 5e with HeLa-H2 cells. Dots represent mean values of independent experiments with error bars representing SD (*n* = 3 per group). Statistical significance was set at *p<*0.05 and was determined with two-sided, unpaired multiple-sample Student's *t-*tests corrected with Holm-Sídák's method for multiple comparisons.


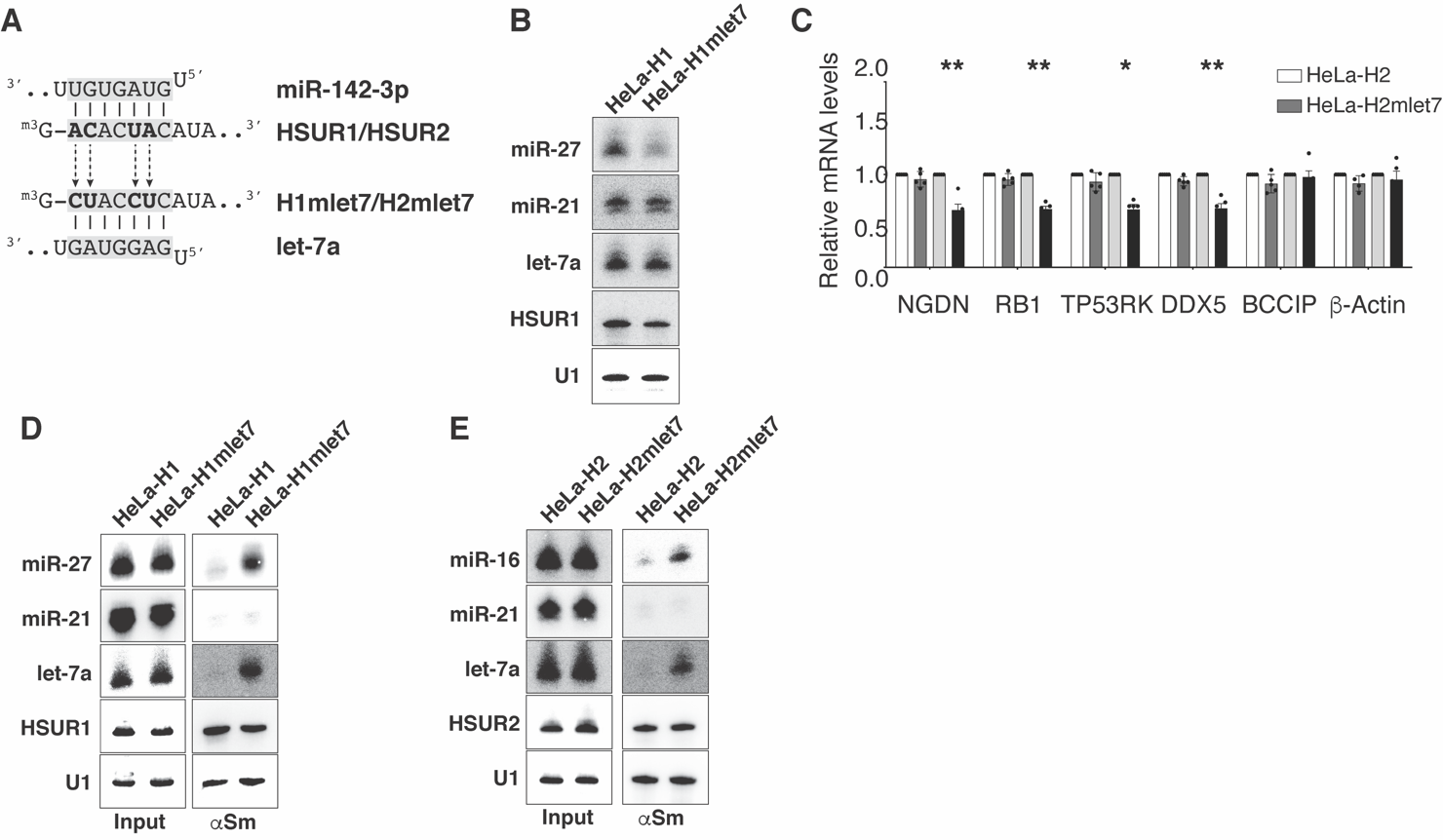


**Supplementary Figure 3. HSUR1 and HSUR2 can be allosterically regulated by let-7a.**

**A)** Partial sequences of the 5′ end of HSUR1 and HSUR2, and miR-142-3p and let-7a seed regions are shown, with residues involved in base-pairing highlighted in gray. Mutated residues are shown in bold. **(B)** Northern blot analysis of equal amounts of total RNA prepared from HeLa-H1 or HeLa cells expressing a mutant version of HSUR1 predicted to bind let-7a at its 5′ end (HeLa-H1mlet7). **(C)** HSUR2 target mRNA levels in HeLa-H2 or HeLa-H2mlet7 cells. Dots represent mean values of independent experiments with error bars representing SD (*n* = 3 per group). Statistical significance was set at *p<*0.05 and was determined with two-sided, unpaired multiple-sample Student's *t-*tests corrected with Holm-Sídák's method for multiple comparisons. ***P*<0.01, **P*<0.05. **(D)** Same as in Figure 7F with HeLa-H1 and HeLa-H1mlet7 cells. **(E)** Same as in Figure 7G with HeLa-H2 and HeLa-H2mlet7 cells.


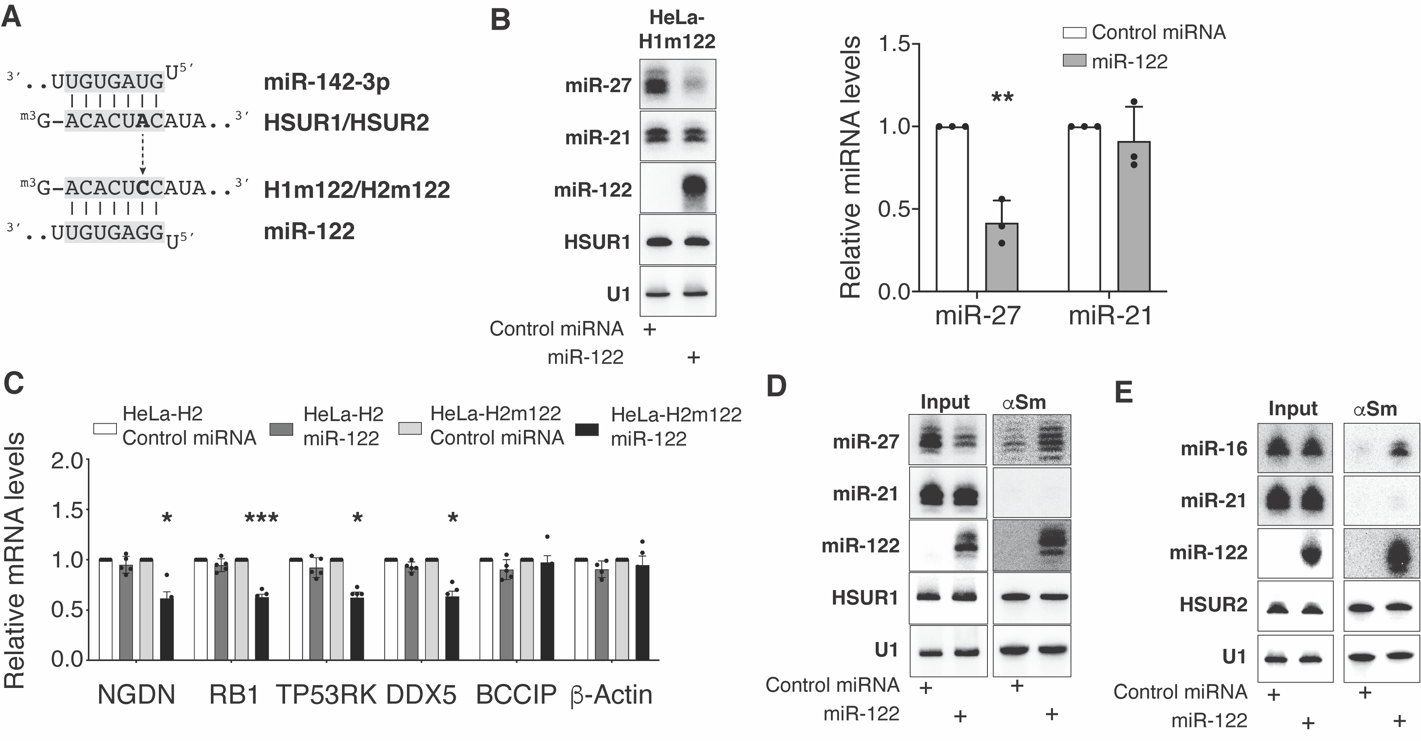


**Supplementary Figure 4. HSUR1 and HSUR2 can be allosterically regulated by transiently expressed miR-122.**

**A)** Partial sequences of the 5′ end of HSUR1 and HSUR2, and miR-142-3p and miR-122 seed regions are shown, with residues involved in base-pairing highlighted in gray. Mutated residues are shown in bold. **(B)** Northern blot analysis of HeLa cells constitutively expressing HSUR1m122 (HeLa-H1m122) transiently transfected with Control miRNA or miR-122. Right, quantification of independent experiments (*n* = 3). U1 snRNA signal was used for normalization. **(C)** As in Figure 2A with HeLa-H2 or HeLa cells constitutively expressing HSUR2m122 (HeLa-H2m122) transiently transfected with Control miRNA or miR-122. **(D)** As in Figure 3 with HeLa-H1m122 cells transiently transfected with Control miRNA or miR-122. **(E)** As in (D) with HeLa-H2m122 cells. For (B) and (C), dots represent mean values of independent experiments with error bars representing SD (*n* = 3 per group). Statistical significance was set at *p<*0.05 and was determined with two-sided, unpaired multiple-sample Student's *t-*tests corrected with Holm-Sídák's method for multiple comparisons. ****P*<0.001, ***P*<0.01, **P*<0.05.
